# [6]Cyclo-2,7-naphthyl­ene: a redetermination

**DOI:** 10.1107/S1600536811023427

**Published:** 2011-06-22

**Authors:** Waka Nakanishi, Jing Yang Xue, Tomoaki Yoshioka, Hiroyuki Isobe

**Affiliations:** a Department of Chemistry, Tohoku University, Aoba-ku, Sendai, 980-8578, Japan

## Abstract

Single crystals of a macrocyclic hydro­carbon, [6]cyclo-2,7-naphthyl­ene ([6]CNAP, C_60_H_36_) were prepared from anthracene melt with a prolonged time for the recrystallization. The crystal of improved quality led to the correction of the space-group assignment to *Cmca* from 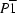 in the original determination [Nakanishi *et al.* (2011 ▶) *Angew. Chem. Int. Ed.* **50**, 5323–5326] and the refinement of anisotropic displacement parameters of all C atoms. The refined mol­ecular structure with *C*_2*h*_ point symmetry indicated that the strain on the naphthyl rings of [6]CNAP is smallest among the congeners. Despite the large macrocyclic structure, mol­ecules are packed in a ubiquitous herringbone motif. A short C—C distance of 3.119 (4) Å was found in the stacking direction, and a short C—H distance of 2.80 Å was found in the inter­columnar contact.

## Related literature

Superior quality crystals of the title compound were obtained by re-optimizing the crystallization conditions. For the synthesis and preceding crystallographic analysis, see: Nakanishi *et al.* (2011 ▶). For the original method of recrystallization, see: Miyahara & Shimizu (2001 ▶). For a review of C—H⋯π contacts in crystals, see: Nishio (2004 ▶).
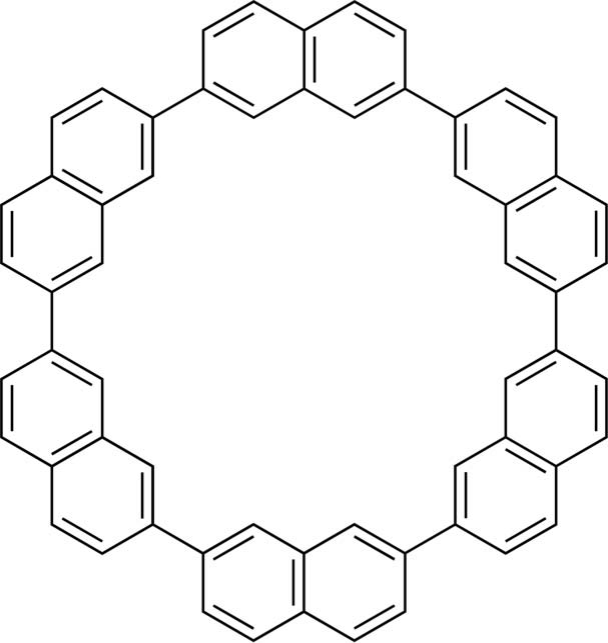


## Experimental

### 

#### Crystal data

C_60_H_36_
*M* *_r_* = 756.89
Orthorhombic, 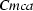

*a* = 34.224 (6) Å
*b* = 7.4629 (14) Å
*c* = 15.131 (3) Å
*V* = 3864.7 (12) Å^3^
*Z* = 4
Mo *K*α radiation
μ = 0.07 mm^−1^
*T* = 100 K
0.40 × 0.12 × 0.06 mm

#### Data collection

Bruker APEXII CCD area-detector diffractometer
Absorption correction: multi-scan (*SADABS*; Sheldrick, 1996 ▶) *T*_min_ = 0.686, *T*_max_ = 0.996
20522 measured reflections
2234 independent reflections
1667 reflections with *I* > 2σ(*I*)
*R*_int_ = 0.035

#### Refinement

*R*[*F* ^2^ > 2σ(*F* ^2^)] = 0.066
*wR*(*F* ^2^) = 0.181
*S* = 1.06
2234 reflections
139 parameters
H-atom parameters constrained
Δρ_max_ = 0.20 e Å^−3^
Δρ_min_ = −0.35 e Å^−3^

### 

Data collection: *APEX2* (Bruker, 2006 ▶); cell refinement: *SAINT* (Bruker, 2004 ▶); data reduction: *SAINT*; program(s) used to solve structure: *SHELXS97* (Sheldrick, 2008 ▶); program(s) used to refine structure: *SHELXL97* (Sheldrick, 2008 ▶); molecular graphics: *ORTEP-3* (Farrugia, 1997 ▶) and *Mercury* (Macrae *et al.*, 2008 ▶); software used to prepare material for publication: *SHELXL97* and *Yadokari-XG 2009* (Kabuto *et al.*, 2009 ▶) and *publCIF* (Westrip, 2010 ▶).

## Supplementary Material

Crystal structure: contains datablock(s) I, global. DOI: 10.1107/S1600536811023427/nr2007sup1.cif

Structure factors: contains datablock(s) I. DOI: 10.1107/S1600536811023427/nr2007Isup2.hkl

Additional supplementary materials:  crystallographic information; 3D view; checkCIF report

## Figures and Tables

**Fig. 1.**
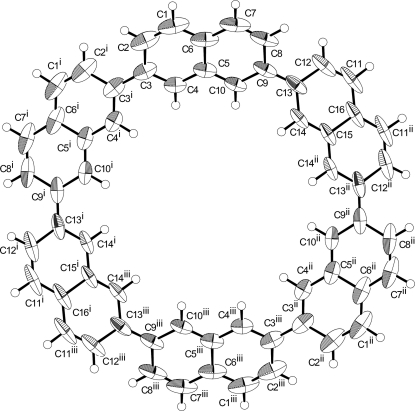
The molecular structure of the title compound with displacement ellipsoids drawn at the 50% probability level. Symmetry codes: (i) x, -y + 2, -z + 1; (ii) -x + 1, y, z; (iii) -x + 1, -y + 2, -z + 1.

**Fig. 2.**
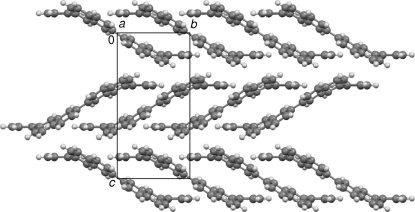
The packing structure of the title compound, viewed along the a axis.

## supplementary crystallographic information

### Comment

Polycyclic aromatic hydrocarbons are important compounds for the development of organic electronics. As new bipolar carrier transport materials for organic light emitting diodes, we recently reported [*n*]cyclo-2,7-naphthylenes ([*n*]CNAP; Nakanishi *et al.*, 2011). The unique macrocyclic structures of [*n*]CNAPs (*n* = 5, 6 and 7) were revealed by X-ray crystallographic analysis of the single crystals, but we deferred detailed discussion of the most abundant compounds, [6]CNAP, because of insufficient quality of available data mainly due to weak reflections from the previous crystals. We now obtained single crystals of [6]CNAP with superior quality by re-optimizing the crystallization conditions and successfully corrected the space group assignment to *Cmca*. The molecular structure of title compound is shown in Fig. 1, and the packing structure is shown in Fig. 2. Most importantly, the refined molecular structure with *C*_2_*_h_* point symmetry shows that [6]CNAP has the smallest deformation in the planar naphthyl rings with the average bend angle of 2.3° which is smaller than 16° and 5° of [5]- and [7]CNAPs, respectively. To form the strain-free macrocycle, the naphthyl rings are twisted alternately with dihedral angles of 33.1 (3)° and 25.6 (4)°. Despite the large macrocyclic structure, molecules are packed in a ubiquitous herringbone motif. A short C—C distance of 3.119 (4) Å was found in the stacking direction, and a short C—H distance of 2.80 Å was found in the intercolumnar contact.

### Experimental

The title compound was synthesized by a nickel promoted coupling reaction of 2,7-dibromonaphthalene and separated as reported in literature (Nakanishi *et al.*, 2011). A single crystal suitable for X-ray crystallographic analysis was obtained by a solid solvent growth method, as reported except that the time for crystal growth was extended: A mixture of anthracene (200 mg) and [6]CNAP (4 mg) was sealed in a glass tube. The whole glass tube was heated at 350 °C for 2 h. The subsequent crystal-growing time at 210 °C was extended from 2 h to 3 h, and the tube was cooled gradually to ambient temperature. A half of the glass tube was then heated at 200 °C to eliminate anthracene and afford crystals of [6]CNAP. For the original method of recrystallization, see: Miyahara & Shimizu (2001).

### Refinement

H atoms were included in calculated positions and treated as riding atoms, with C—H = 0.95 Å (aromatic) and *U*_iso_(H) = 1.2*U*_eq_(C).

### Crystal data

**Table:**

C_60_H_36_	*F*(000) = 1584
*M**_r_* = 756.89	*D*_x_ = 1.301 Mg m^−^^3^
Orthorhombic, *C**m**c**a*	Mo *K*α radiation, λ = 0.71073 Å
Hall symbol: -C 2bc 2	Cell parameters from 5445 reflections
*a* = 34.224 (6) Å	θ = 2.4–27.2°
*b* = 7.4629 (14) Å	µ = 0.07 mm^−^^1^
*c* = 15.131 (3) Å	*T* = 100 K
*V* = 3864.7 (12) Å^3^	Plate, colourless
*Z* = 4	0.40 × 0.12 × 0.06 mm

### Data collection

**Table:**

Bruker APEXII CCD area-detector diffractometer	2234 independent reflections
Radiation source: Bruker TXS fine-focus rotating anode	1667 reflections with *I* > 2σ(*I*)
Bruker Helios multilayer confocal mirror	*R*_int_ = 0.035
Detector resolution: 8.333 pixels mm^-1^	θ_max_ = 27.5°, θ_min_ = 2.4°
φ and ω scans	*h* = −43→43
Absorption correction: multi-scan (*SADABS*; Sheldrick, 1996)	*k* = −9→9
*T*_min_ = 0.686, *T*_max_ = 0.996	*l* = −19→19
20522 measured reflections	

### Refinement

**Table:**

Refinement on *F*^2^	Primary atom site location: structure-invariant direct methods
Least-squares matrix: full	Secondary atom site location: difference Fourier map
*R*[*F*^2^ > 2σ(*F*^2^)] = 0.066	Hydrogen site location: inferred from neighbouring sites
*wR*(*F*^2^) = 0.181	H-atom parameters constrained
*S* = 1.06	*w* = 1/[σ^2^(*F*_o_^2^) + (0.0565*P*)^2^ + 7.0687*P*] where *P* = (*F*_o_^2^ + 2*F*_c_^2^)/3
2234 reflections	(Δ/σ)_max_ < 0.001
139 parameters	Δρ_max_ = 0.20 e Å^−^^3^
0 restraints	Δρ_min_ = −0.35 e Å^−^^3^

### Special details

**Table:**

Geometry. All e.s.d.'s (except the e.s.d. in the dihedral angle between two l.s. planes) are estimated using the full covariance matrix. The cell e.s.d.'s are taken into account individually in the estimation of e.s.d.'s in distances, angles and torsion angles; correlations between e.s.d.'s in cell parameters are only used when they are defined by crystal symmetry. An approximate (isotropic) treatment of cell e.s.d.'s is used for estimating e.s.d.'s involving l.s. planes.
Refinement. Refinement of *F*^2^ against ALL reflections. The weighted *R*-factor *wR* and goodness of fit *S* are based on *F*^2^, conventional *R*-factors *R* are based on *F*, with *F* set to zero for negative *F*^2^. The threshold expression of *F*^2^ > σ(*F*^2^) is used only for calculating *R*-factors(gt) *etc*. and is not relevant to the choice of reflections for refinement. *R*-factors based on *F*^2^ are statistically about twice as large as those based on *F*, and *R*- factors based on ALL data will be even larger.

### Fractional atomic coordinates and isotropic or equivalent isotropic displacement parameters (Å^2^)

**Table:**

	*x*	*y*	*z*	*U*_iso_*/*U*_eq_	
C1	0.28550 (9)	0.7008 (6)	0.37933 (17)	0.0855 (12)	
H1	0.2623	0.6634	0.3504	0.103*	
C2	0.28488 (8)	0.8521 (5)	0.43007 (18)	0.0805 (11)	
H2	0.2610	0.9154	0.4371	0.097*	
C3	0.31917 (7)	0.9174 (4)	0.47282 (14)	0.0599 (7)	
C4	0.35317 (7)	0.8204 (3)	0.46178 (14)	0.0521 (6)	
H3	0.3764	0.8613	0.4897	0.062*	
C5	0.35467 (8)	0.6627 (4)	0.41049 (14)	0.0546 (7)	
C6	0.32004 (9)	0.5987 (5)	0.36887 (15)	0.0705 (9)	
C7	0.32231 (11)	0.4362 (5)	0.32059 (16)	0.0842 (12)	
H4	0.2997	0.3918	0.2916	0.101*	
C8	0.35645 (11)	0.3425 (4)	0.31496 (15)	0.0777 (11)	
H5	0.3570	0.2332	0.2828	0.093*	
C9	0.39145 (9)	0.4048 (3)	0.35623 (14)	0.0599 (8)	
C10	0.38962 (8)	0.5646 (3)	0.40197 (13)	0.0518 (6)	
H6	0.4127	0.6100	0.4286	0.062*	
C11	0.46345 (13)	0.0185 (3)	0.35550 (17)	0.0872 (12)	
H7	0.4628	−0.1088	0.3562	0.105*	
C12	0.42913 (13)	0.1103 (4)	0.35358 (16)	0.0781 (11)	
H8	0.4052	0.0460	0.3522	0.094*	
C13	0.42846 (10)	0.3024 (3)	0.35367 (14)	0.0602 (8)	
C14	0.46407 (8)	0.3892 (3)	0.35413 (14)	0.0542 (7)	
H9	0.4643	0.5165	0.3534	0.065*	
C15	0.5000	0.2979 (4)	0.35555 (19)	0.0556 (10)	
C16	0.5000	0.1066 (4)	0.3565 (2)	0.0708 (13)	

### Atomic displacement parameters (Å^2^)

**Table:**

	*U*^11^	*U*^22^	*U*^33^	*U*^12^	*U*^13^	*U*^23^
C1	0.0610 (18)	0.162 (4)	0.0339 (13)	−0.048 (2)	−0.0068 (12)	0.0195 (17)
C2	0.0528 (16)	0.151 (3)	0.0375 (13)	−0.0189 (18)	−0.0027 (11)	0.0297 (17)
C3	0.0481 (13)	0.100 (2)	0.0322 (11)	−0.0087 (13)	−0.0009 (9)	0.0234 (11)
C4	0.0553 (14)	0.0695 (15)	0.0314 (10)	−0.0175 (12)	−0.0064 (9)	0.0157 (10)
C5	0.0663 (16)	0.0706 (16)	0.0270 (10)	−0.0286 (13)	−0.0066 (10)	0.0130 (10)
C6	0.0719 (18)	0.114 (2)	0.0260 (10)	−0.0495 (17)	−0.0031 (10)	0.0138 (13)
C7	0.094 (2)	0.129 (3)	0.0301 (12)	−0.074 (2)	−0.0006 (13)	0.0028 (15)
C8	0.116 (3)	0.088 (2)	0.0281 (11)	−0.071 (2)	0.0065 (14)	−0.0042 (12)
C9	0.098 (2)	0.0553 (14)	0.0264 (10)	−0.0412 (15)	−0.0006 (11)	0.0023 (9)
C10	0.0732 (16)	0.0537 (13)	0.0286 (10)	−0.0308 (12)	−0.0056 (10)	0.0062 (9)
C11	0.197 (4)	0.0250 (12)	0.0396 (13)	−0.0237 (18)	0.0074 (18)	−0.0075 (10)
C12	0.160 (3)	0.0402 (14)	0.0340 (12)	−0.0427 (18)	0.0088 (16)	−0.0048 (10)
C13	0.114 (2)	0.0398 (12)	0.0267 (10)	−0.0318 (14)	0.0039 (12)	−0.0045 (9)
C14	0.106 (2)	0.0247 (9)	0.0321 (10)	−0.0139 (11)	0.0003 (11)	−0.0036 (8)
C15	0.114 (3)	0.0230 (14)	0.0300 (14)	0.000	0.000	−0.0045 (11)
C16	0.157 (4)	0.0237 (15)	0.0318 (16)	0.000	0.000	−0.0046 (12)

### Geometric parameters (Å, °)

**Table:**

C1—C2	1.365 (5)	C8—H5	0.9500
C1—C6	1.416 (5)	C9—C10	1.381 (3)
C1—H1	0.9500	C9—C13	1.480 (4)
C2—C3	1.426 (4)	C10—H6	0.9500
C2—H2	0.9500	C11—C12	1.360 (5)
C3—C4	1.380 (3)	C11—C16	1.413 (4)
C3—C3^i^	1.482 (6)	C11—H7	0.9500
C4—C5	1.410 (4)	C12—C13	1.434 (4)
C4—H3	0.9500	C12—H8	0.9500
C5—C10	1.408 (4)	C13—C14	1.380 (4)
C5—C6	1.425 (3)	C14—C15	1.406 (3)
C6—C7	1.418 (5)	C14—H9	0.9500
C7—C8	1.364 (5)	C15—C14^ii^	1.406 (3)
C7—H4	0.9500	C15—C16	1.428 (4)
C8—C9	1.429 (4)	C16—C11^ii^	1.413 (4)
			
C2—C1—C6	121.4 (3)	C10—C9—C8	117.5 (3)
C2—C1—H1	119.3	C10—C9—C13	119.9 (2)
C6—C1—H1	119.3	C8—C9—C13	122.6 (3)
C1—C2—C3	121.7 (3)	C9—C10—C5	122.3 (2)
C1—C2—H2	119.2	C9—C10—H6	118.9
C3—C2—H2	119.2	C5—C10—H6	118.9
C4—C3—C2	117.4 (3)	C12—C11—C16	122.0 (2)
C4—C3—C3^i^	120.22 (15)	C12—C11—H7	119.0
C2—C3—C3^i^	122.4 (2)	C16—C11—H7	119.0
C3—C4—C5	122.3 (2)	C11—C12—C13	121.2 (3)
C3—C4—H3	118.8	C11—C12—H8	119.4
C5—C4—H3	118.8	C13—C12—H8	119.4
C10—C5—C4	121.0 (2)	C14—C13—C12	117.1 (3)
C10—C5—C6	119.4 (3)	C14—C13—C9	120.9 (2)
C4—C5—C6	119.5 (3)	C12—C13—C9	122.0 (3)
C1—C6—C7	124.3 (3)	C13—C14—C15	123.0 (2)
C1—C6—C5	117.7 (3)	C13—C14—H9	118.5
C7—C6—C5	118.0 (3)	C15—C14—H9	118.5
C8—C7—C6	121.2 (3)	C14^ii^—C15—C14	122.0 (3)
C8—C7—H4	119.4	C14^ii^—C15—C16	118.99 (13)
C6—C7—H4	119.4	C14—C15—C16	118.99 (13)
C7—C8—C9	121.6 (3)	C11^ii^—C16—C11	124.5 (4)
C7—C8—H5	119.2	C11^ii^—C16—C15	117.71 (19)
C9—C8—H5	119.2	C11—C16—C15	117.71 (19)
			
C6—C1—C2—C3	1.9 (4)	C4—C5—C10—C9	176.39 (19)
C1—C2—C3—C4	−0.7 (4)	C6—C5—C10—C9	−1.7 (3)
C1—C2—C3—C3^i^	179.2 (3)	C16—C11—C12—C13	−0.9 (4)
C2—C3—C4—C5	0.1 (3)	C11—C12—C13—C14	1.2 (4)
C3^i^—C3—C4—C5	−179.8 (2)	C11—C12—C13—C9	−176.8 (2)
C3—C4—C5—C10	−178.7 (2)	C10—C9—C13—C14	−33.1 (3)
C3—C4—C5—C6	−0.6 (3)	C8—C9—C13—C14	148.8 (2)
C2—C1—C6—C7	177.0 (2)	C10—C9—C13—C12	144.8 (2)
C2—C1—C6—C5	−2.3 (4)	C8—C9—C13—C12	−33.2 (3)
C10—C5—C6—C1	179.8 (2)	C12—C13—C14—C15	−0.8 (3)
C4—C5—C6—C1	1.7 (3)	C9—C13—C14—C15	177.2 (2)
C10—C5—C6—C7	0.4 (3)	C13—C14—C15—C14^ii^	178.65 (17)
C4—C5—C6—C7	−177.7 (2)	C13—C14—C15—C16	0.1 (4)
C1—C6—C7—C8	−178.5 (2)	C12—C11—C16—C11^ii^	−177.8 (2)
C5—C6—C7—C8	0.8 (4)	C12—C11—C16—C15	0.1 (4)
C6—C7—C8—C9	−0.9 (4)	C14^ii^—C15—C16—C11^ii^	−0.3 (4)
C7—C8—C9—C10	−0.3 (3)	C14—C15—C16—C11^ii^	178.3 (2)
C7—C8—C9—C13	177.8 (2)	C14^ii^—C15—C16—C11	−178.3 (2)
C8—C9—C10—C5	1.6 (3)	C14—C15—C16—C11	0.3 (4)
C13—C9—C10—C5	−176.57 (19)		

Symmetry codes: (i) *x*, −*y*+2, −*z*+1; (ii) −*x*+1, *y*, *z*.
